# Supplementary material for: Four-dimensional hydrogel dressing adaptable to the urethral microenvironment for scarless urethral reconstruction
Source: Nat Commun. 2023 Nov 22;14:7632. doi: 10.1038/s41467-023-43421-w (PMC10665446; doi:10.1038/s41467-023-43421-w)
Supplement: Supplementary file 1 — Supplementary Information [file 41467_2023_43421_MOESM1_ESM.pdf]

Supplementary Information

Four-dimensional Hydrogel Dressing Adaptable to the Urethral Microenvironment for Scarless Urethral Reconstruction

Yujie Hua[1,2,5], Kai Wang[3,5], Yingying Huo[2,5], Yaping Zhuang[4], Yuhui Wang[1], Wenzhuo Fang[1], Yuyan Sun[2], Guangdong Zhou[2], Qiang Fu[1]*, Wenguo Cui[4]* & Kaile Zhang[1]*

[1]Department of Urology, Shanghai Sixth People's Hospital Affiliated to Shanghai Jiao Tong University School of Medicine, Shanghai Jiao Tong University, Shanghai 200233, P. R. China.

[2]Department of Plastic and Reconstructive Surgery, Shanghai Ninth People's Hospital Affiliated to Shanghai Jiao Tong University School of Medicine, Shanghai Key Laboratory of Tissue Engineering, Shanghai 200011, P. R. China.

[3]Clinical Research Center, Shanghai Chest Hospital, Shanghai Jiao Tong University School of Medicine, Shanghai 200030, P. R. China.

[4]Shanghai Institute of Traumatology and Orthopaedics, Shanghai Key Laboratory for Prevention and Treatment of Bone and Joint Diseases, Ruijin Hospital, Shanghai Jiao Tong University School of Medicine, Shanghai 200025, P. R. China.

[5]These authors contributed equally: Yujie Hua, Kai Wang, Yingying Huo.

*Corresponding author. E-mail: great_z0313@sjtu.edu.cn (K.Z.); jamesqfu@126.com (Q.F.); wgcui@sjtu.edu.cn (W.C.)

This file includes: Supplementary Fig. 1 to 17.

Supplementary Figures

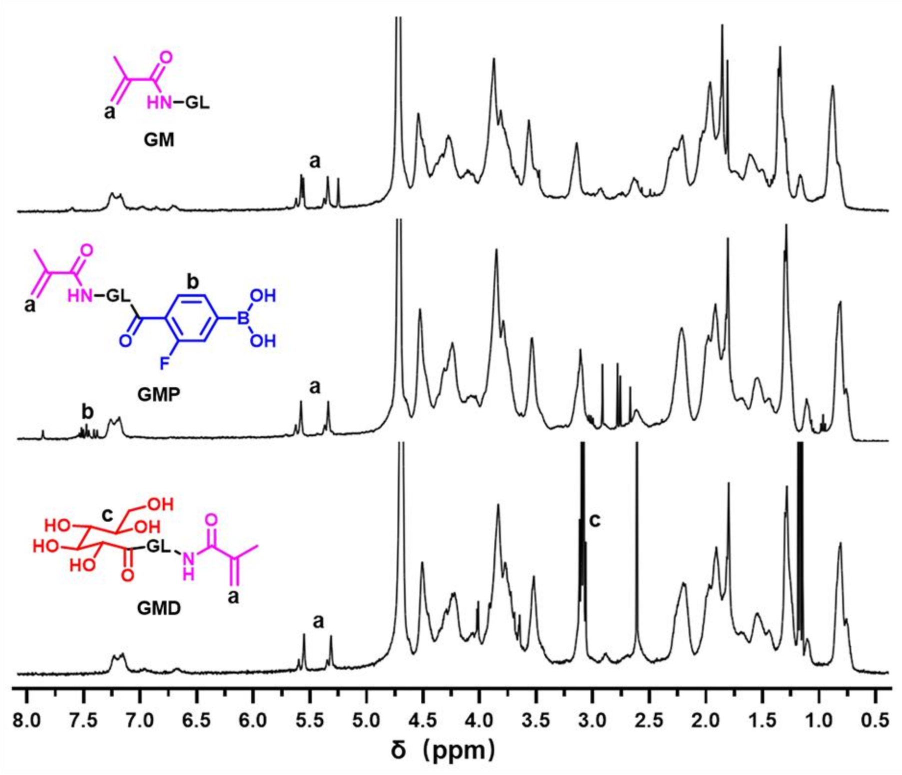

Supplementary Fig. 1. ^{1}H NMR spectral characterization of gelatin methacryloyl (**GM**), fluorophenylboronic acid (FPBA)-modified gelatin methacryloyl (**GMP**), and *cis*-diol-modified gelatin methacryloyl (**GMD**).

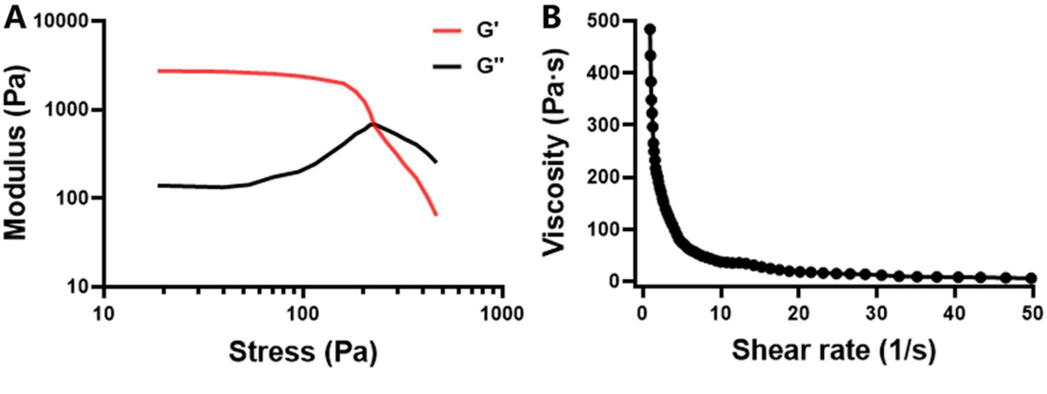

Supplementary Fig. 2. **A**) Representative strain sweep rheological plots of ***GMPD-hv*** hydrogels. **B**) Viscosity and shear-thinning behavior of ***GMPD-hv*** hydrogels. n = 4 independent samples.

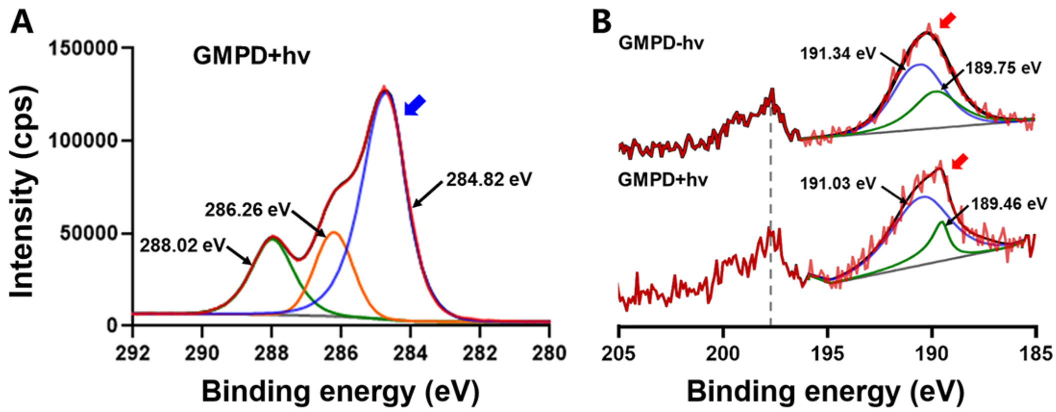

Supplementary Fig. 3. A) The C(1s) *XPS* regions of **GMPD+*hv*** hydrogel samples. Intensity (cps): count per second. Red line: C(1s) characteristic speak; green line: C=O (288.02 eV); orange line: C-O (286.26 eV); blue line: C-C (284.82 eV); blue arrows represent C(1s) regions. **B**) The B(1s) *XPS* regions of **GMPD+*hv*** hydrogel samples. Red line: B(1s) characteristic speak; blue line: B-O (191.03 eV); green line: B-C (189.46 eV); red arrows represent B(1s) regions.

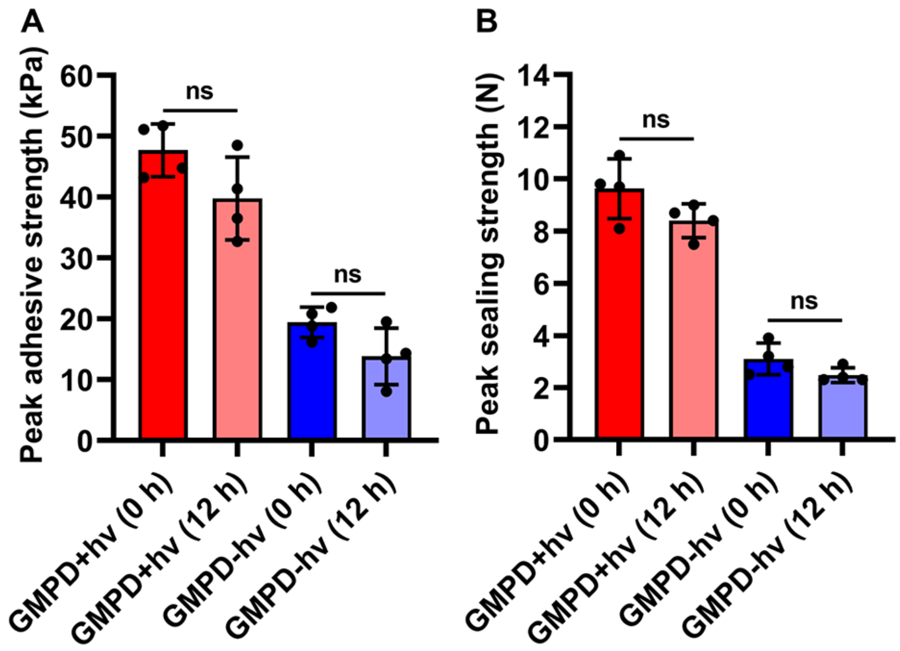

Supplementary Fig. 4. The statistical data of standard lap shear (**A**) and incision sealing (**B**) tests showing the *ex vivo* adhesive performance after 0 and 12 hours. n = 4 independent samples. Data are presented as mean ± SD. All error bars represent SD. p values calculated using one-tailed unpaired t-test. ns = no significance. Source data are provided as a Source

Data file.

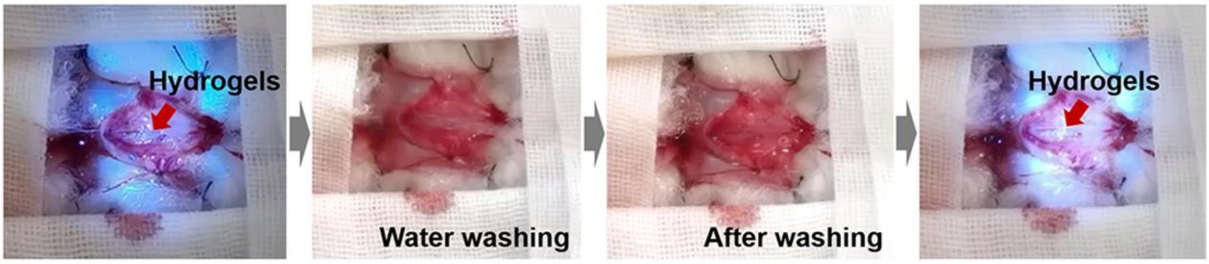

Supplementary Fig. 5. Representative photographs showing the resistance to water washing at approximately 10 kPa water pressure in the urethral defect model. Red arrows represent the adhesive hydrogels stained by green fluorescence. n = 3 independent samples.

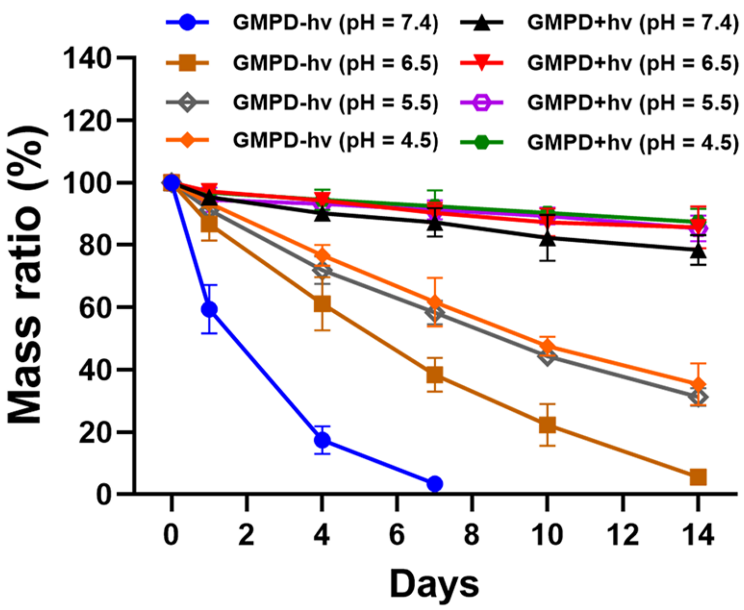

Supplementary Fig. 6. The *in vitro* degradation curves of **GMPD-*hv*** and **GMPD+*hv*** hydrogels in neutral (pH = 7.4) or acidic solutions (pH = 4.5-6.5). n = 4 independent samples. Data are presented as mean ± SD. All error bars represent SD. Source data are provided as a Source Data file.

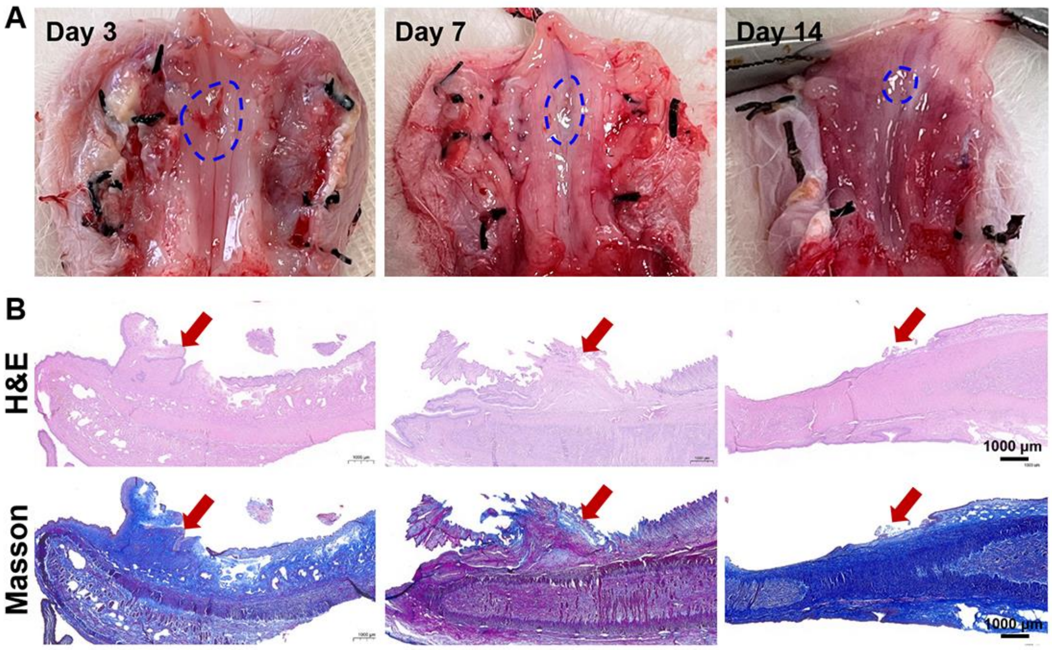

Supplementary Fig. 7. A, B) Representative photographs (**A**) and histological examinations of H&E and Masson's trichrome staining (**B**) of the remaining **GMPD** hydrogels in the urethral defect model after 3-, 7-, 14-days surgery. Blue circles and red arrows represent the remaining hydrogels. n = 3 biologically independent samples.

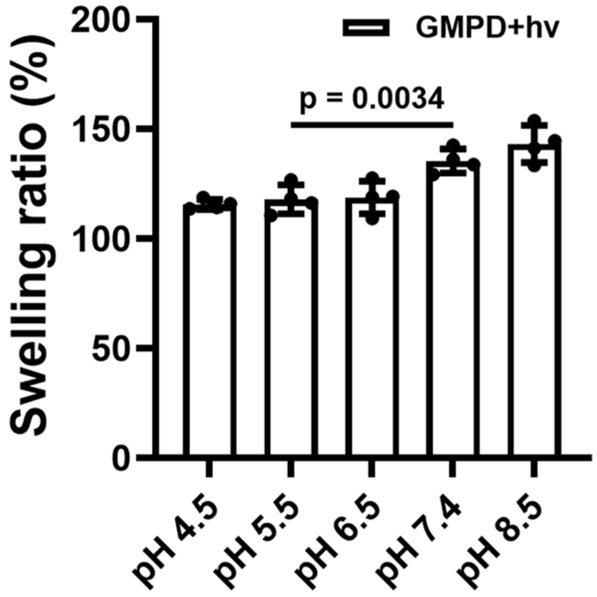

Supplementary Fig. 8. The *in vitro* swelling ratio of **GMPD+*hv*** hydrogels in different pH values ranging from 4.5-8.5. n = 4 independent samples. Data are presented as mean ± SD. All error bars represent SD. p values calculated using one-tailed unpaired t-test. Source data are provided as a Source Data file.

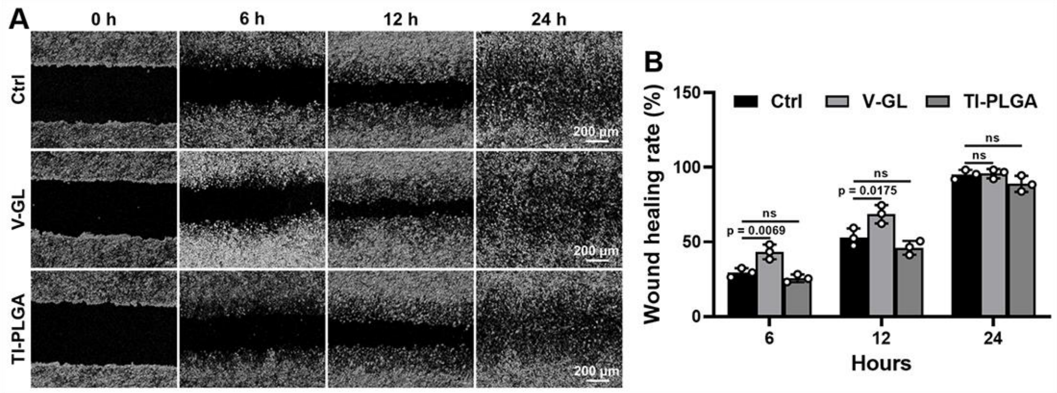

Supplementary Fig. 9. **A**) Observation of the endothelial cell scratch assay after 0, 6, 12, and 24 hours in the **V-GM**, **TI-PLGA**, and control (**Ctrl**) groups. **B**) Statistical analyses of the corresponding wound healing rates. n = 3 biologically independent samples. Data are presented as mean ± SD. All error bars represent SD. p values calculated using one-tailed unpaired t-test. ns = no significance. Source data are provided as a Source Data file.

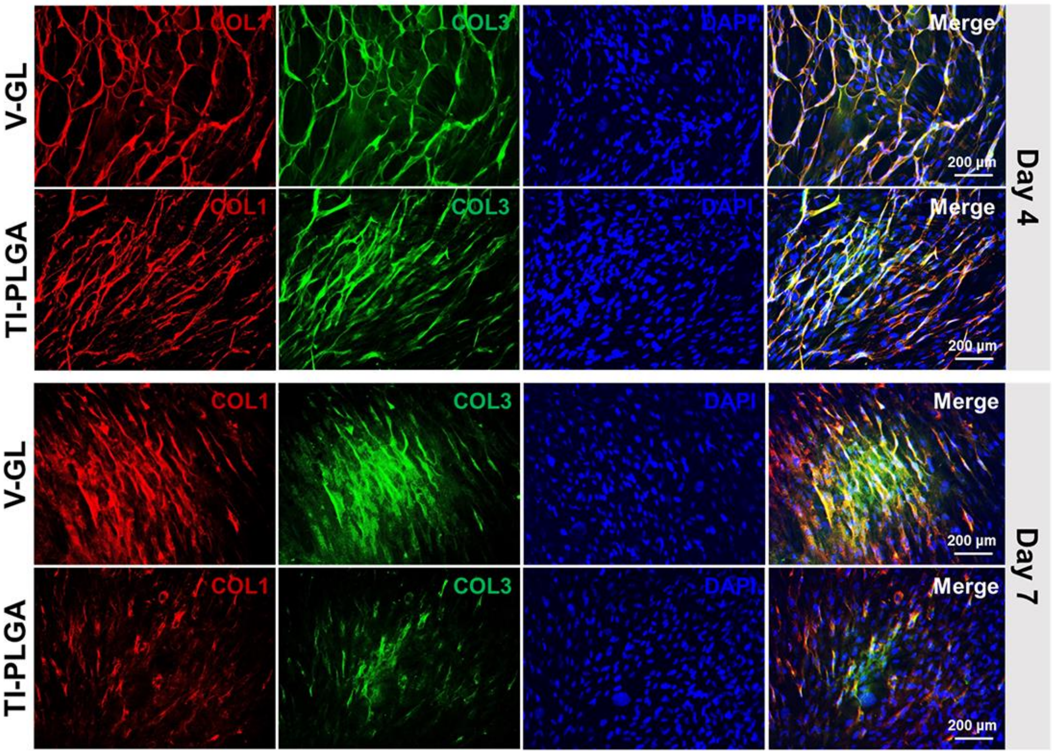

Supplementary Fig. 10. Immunofluorescence staining of COL1 (red), COL3 (green), and cell nuclei (DAPI, blue) for fibroblast marker expression after 4- and 7-days culture in the **V-GM** and **TI-PLGA** groups. n = 3 biologically independent samples.

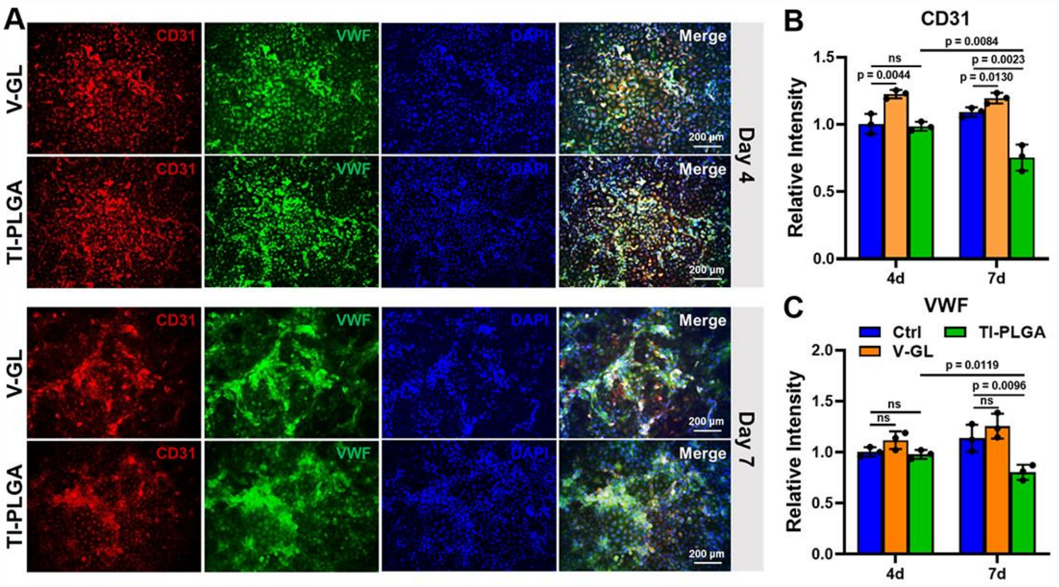

Supplementary Fig. 11. A) Immunofluorescence staining of CD31 (red), VWF (green), and cell nuclei (DAPI, blue) for endothelial cell marker expression after 4- and 7-days culture in the **V-GM** and **TI-PLGA** groups. **B, C**) Comparative endothelial cell expression levels (*CD31* and *VWF*) after 4- and 7-days culture in the **V-GM**, **TI-PLGA**, and control (**Ctrl**) groups. n = 3 biologically independent samples. Data are presented as mean ± SD. All error bars represent SD. p values calculated using one-tailed unpaired t-test. ns = no significance. Source data are provided as a Source Data file.

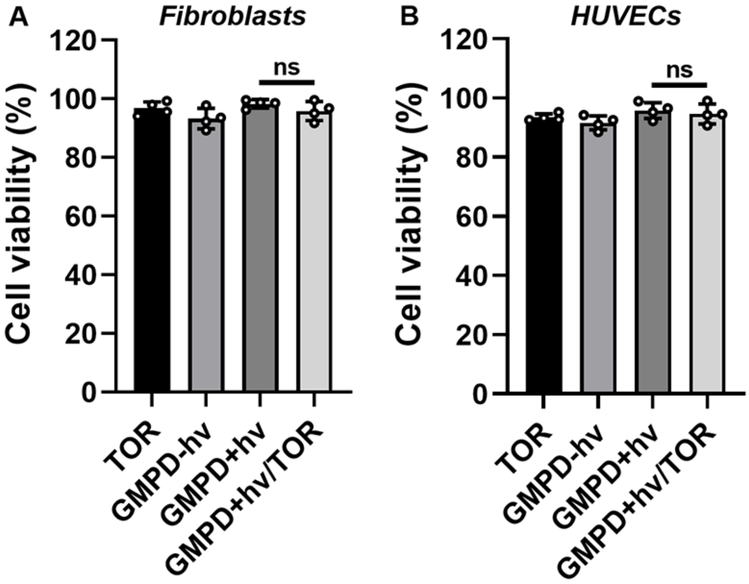

Supplementary Fig. 12. A, B) The CCK-8 data of 1% w/v extract solution of **TOR**

microspheres, **GMPD-*hv***, **GMPD+*hv***, and **GMPD+*hv*/TOR** hydrogels after 24-hours culture for both fibroblasts (**A**) and HUVECs (**B**). n = 4 biologically independent samples. Data are presented as mean ± SD. All error bars represent SD. p values calculated using one-tailed unpaired t-test. ns = no significance. Source data are provided as a Source Data file.

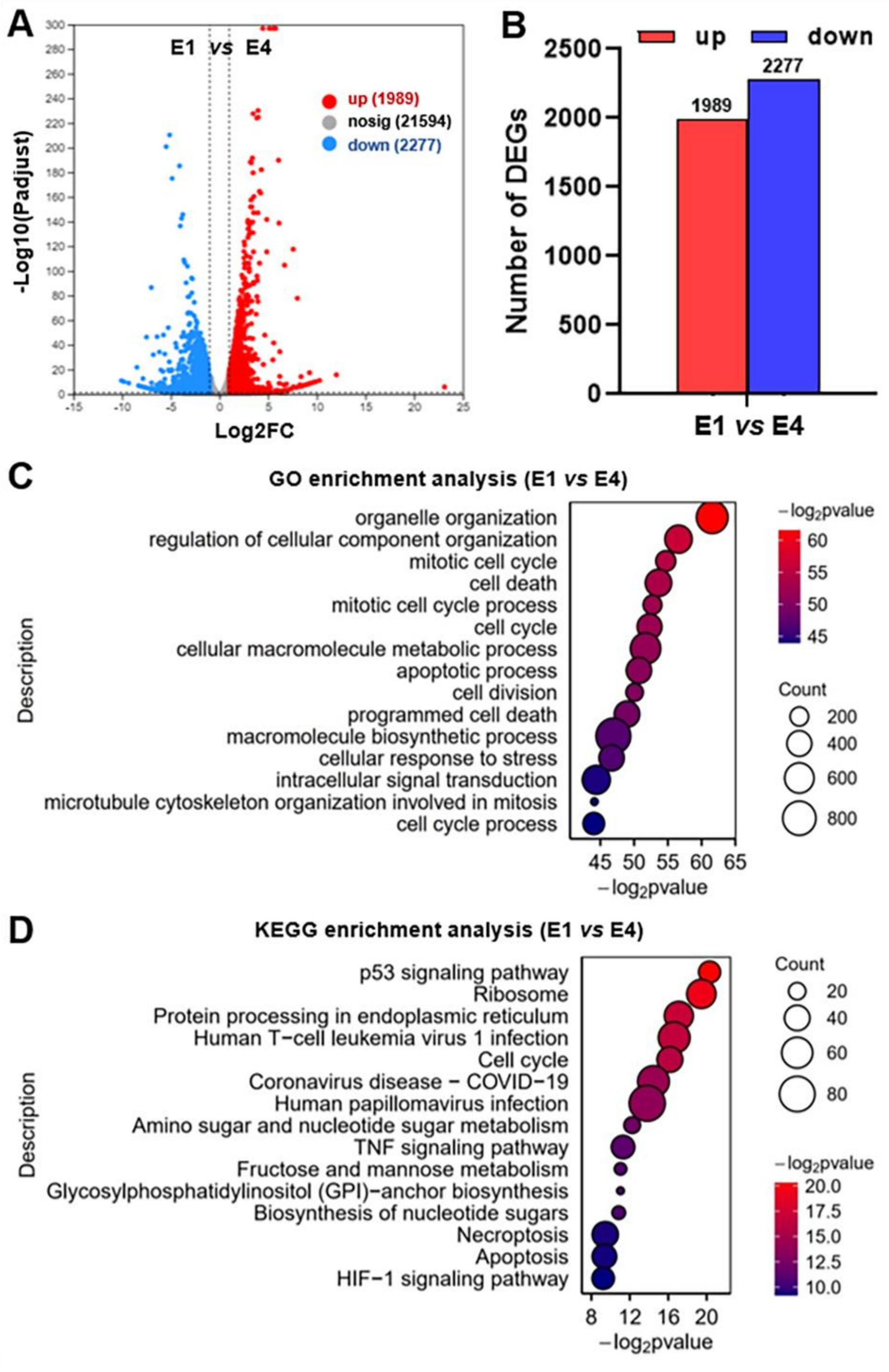

Supplementary Fig. 13. A, B) Volcano plot (**A**) and statistical data (**B**) of endothelial cell

DEGs analyzed between the E1 and E4 groups. **C, D**) The Gene Ontology (GO, **C**) and Kyoto Encyclopedia of Genes and Genomes (KEGG, **D**) enrichment analyses of endothelial cell DEGs after mRNA sequencing in **TOR**-functionalized hydrogels between the E1 and E4 groups, including the top 15 representative upregulated or downregulated signaling pathways.

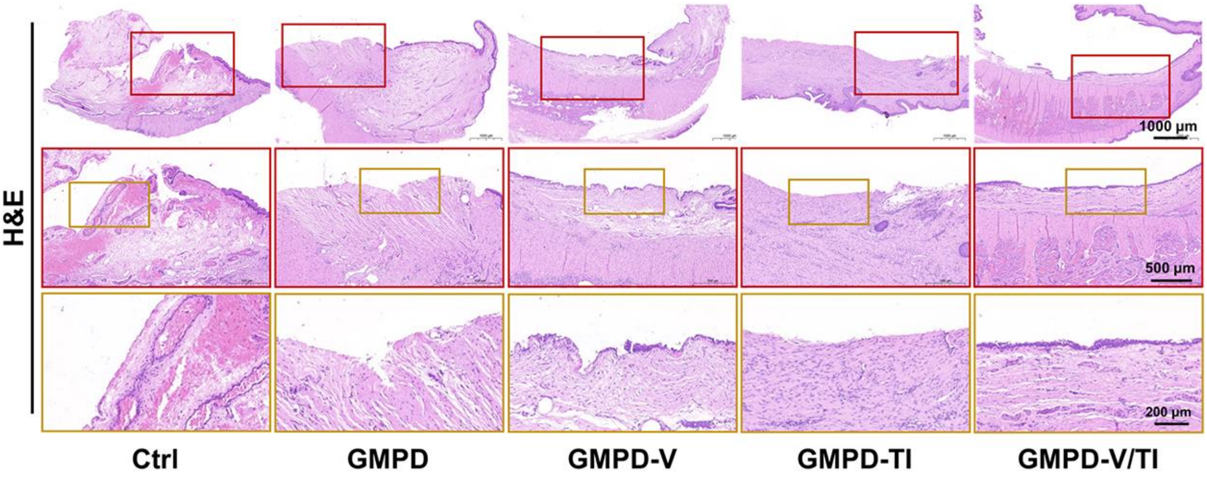

Supplementary Fig. 14. The magnified H&E staining of the rabbit urethral canal in the **GMPD**, **GMPD-V**, **GMPD-TI**, **GMPD-V/TI**, and control (**Ctrl**) groups after 8-weeks surgery. n = 3 biologically independent samples.

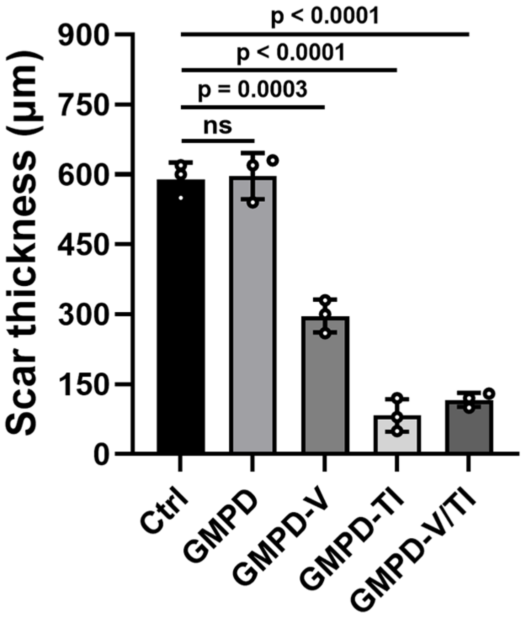

Supplementary Fig. 15. The semi-quantitative data of Masson's trichrome staining of the scar thickness in the **GMPD**, **GMPD-V**, **GMPD-TI**, **GMPD-V/TI**, and control (**Ctrl**) groups

after 8-weeks surgery. n = 3 biologically independent samples. Data are presented as mean ± SD. All error bars represent SD. p values calculated using one-tailed unpaired t-test. ns = no significance. Source data are provided as a Source Data file.

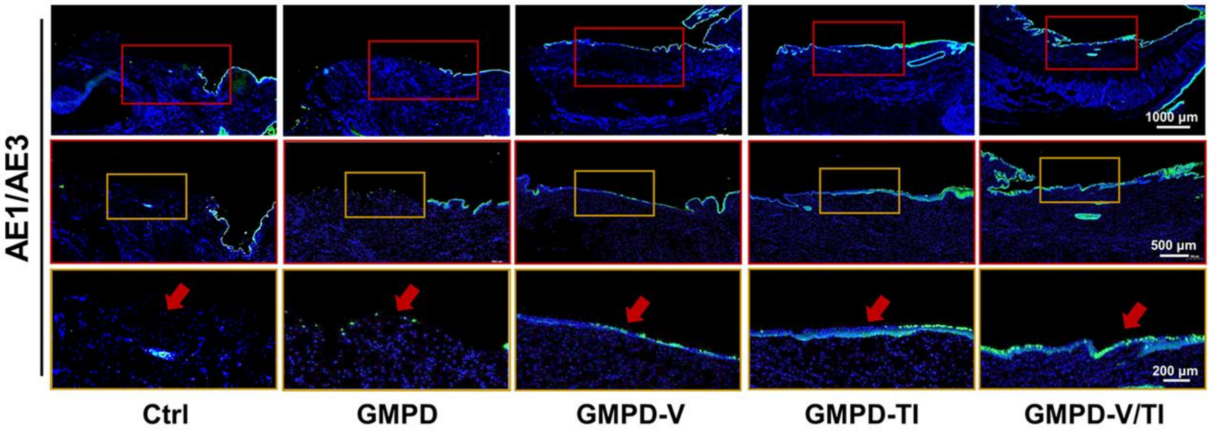

Supplementary Fig. 16. The magnified immunofluorescence staining of the rabbit urethral canal for evaluating epithelialization (AE1/AE3) after different treatments (i.e., **GMPD**, **GMPD-V**, **GMPD-TI**, **GMPD-V/TI**, and **Ctrl** groups) for 8 weeks. n = 3 biologically independent samples.

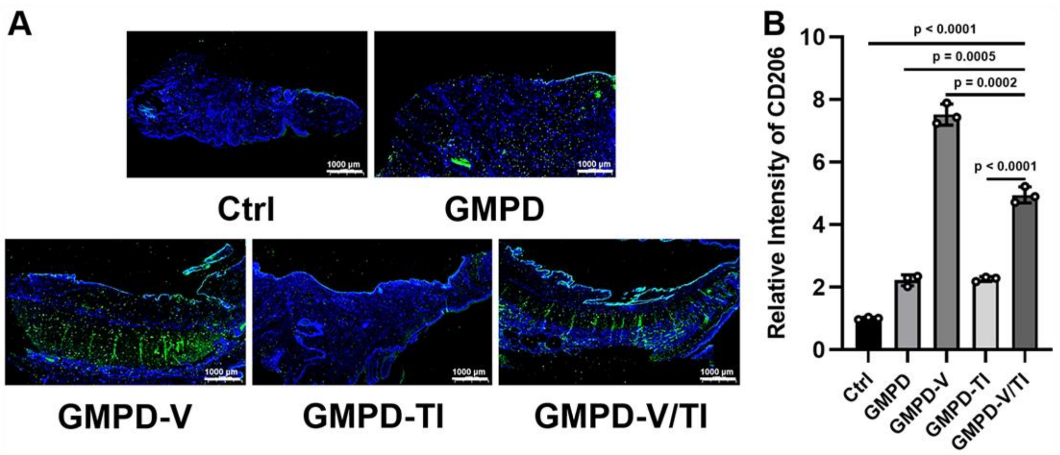

Supplementary Fig. 17. A, B) Immunofluorescence staining (**A**) and quantitative expression level (**B**) of the rabbit urethral canal for evaluating the M2 macrophages (CD206) after different treatments (i.e., **GMPD**, **GMPD-V**, **GMPD-TI**, **GMPD-V/TI**, and **Ctrl** groups) for 8 weeks. n = 3 biologically independent samples. Data are presented as mean ± SD. All error bars represent SD. p values calculated using one-tailed unpaired t-test. Source data are provided as a Source Data file.
