# Supplementary material for: Four-dimensional hydrogel dressing adaptable to the urethral microenvironment for scarless urethral reconstruction
Source: Nat Commun. 2023 Nov 22;14:7632. doi: 10.1038/s41467-023-43421-w (PMC10665446; doi:10.1038/s41467-023-43421-w)
Supplement: Supplementary file 2 — Reporting Summary [file 41467_2023_43421_MOESM2_ESM.pdf]

nature portfolio

Corresponding author(s): Wenguo Cui, Qiang Fu, Kaile Zhang

Last updated by author(s): Nov 8, 2023

Reporting Summary

Nature Portfolio wishes to improve the reproducibility of the work that we publish. This form provides structure for consistency and transparency in reporting. For further information on Nature Portfolio policies, see our Editorial Policies and the Editorial Policy Checklist.

Statistics

For all statistical analyses, confirm that the following items are present in the figure legend, table legend, main text, or Methods section.

n/a	Confirmed	
☐	☒	The exact sample size (*n*) for each experimental group/condition, given as a discrete number and unit of measurement
☐	☒	A statement on whether measurements were taken from distinct samples or whether the same sample was measured repeatedly
☐	☒	The statistical test(s) used AND whether they are one- or two-sided<br>*Only common tests should be described solely by name; describe more complex techniques in the Methods section.*
☒	☐	A description of all covariates tested
☒	☐	A description of any assumptions or corrections, such as tests of normality and adjustment for multiple comparisons
☐	☒	A full description of the statistical parameters including central tendency (e.g. means) or other basic estimates (e.g. regression coefficient) AND variation (e.g. standard deviation) or associated estimates of uncertainty (e.g. confidence intervals)
☐	☒	For null hypothesis testing, the test statistic (e.g. *F*, *t*, *r*) with confidence intervals, effect sizes, degrees of freedom and *P* value noted<br>*Give P values as exact values whenever suitable.*
☒	☐	For Bayesian analysis, information on the choice of priors and Markov chain Monte Carlo settings
☒	☐	For hierarchical and complex designs, identification of the appropriate level for tests and full reporting of outcomes
☒	☐	Estimates of effect sizes (e.g. Cohen's *d*, Pearson's *r*), indicating how they were calculated

Our web collection on statistics for biologists contains articles on many of the points above.

Software and code

Policy information about availability of computer code

Data collection	ImageJ1.8.0 software was used in this study.
Data analysis	The statistical data of relative fluorescent intensity were analyzed using ImageJ software.

For manuscripts utilizing custom algorithms or software that are central to the research but not yet described in published literature, software must be made available to editors and reviewers. We strongly encourage code deposition in a community repository (e.g. GitHub). See the Nature Portfolio guidelines for submitting code & software for further information.

Data

Policy information about availability of data

All manuscripts must include a data availability statement. This statement should provide the following information, where applicable:

- Accession codes, unique identifiers, or web links for publicly available datasets
- A description of any restrictions on data availability
- For clinical datasets or third party data, please ensure that the statement adheres to our policy

The authors declare that all data supporting of results in this study are available within the paper and its Supplementary Information, or from the corresponding authors upon request. Source data are provided with this paper.

Research involving human participants, their data, or biological material

Policy information about studies with human participants or human data. See also policy information about sex, gender (identity/presentation), and sexual orientation and race, ethnicity and racism.

Reporting on sex and gender	No human participates were used.
Reporting on race, ethnicity, or other socially relevant groupings	No additional information.
Population characteristics	No additional information.
Recruitment	No additional information.
Ethics oversight	No additional information.

Note that full information on the approval of the study protocol must also be provided in the manuscript.

Field-specific reporting

Please select the one below that is the best fit for your research. If you are not sure, read the appropriate sections before making your selection.

[x] Life sciences [ ] Behavioural & social sciences [ ] Ecological, evolutionary & environmental sciences

For a reference copy of the document with all sections, see nature.com/documents/nr-reporting-summary-flat.pdf

Life sciences study design

All studies must disclose on these points even when the disclosure is negative.

Sample size	All data (n = 3) are presented as the means ± SDs. Differences between the values were evaluated using one-way analysis of variance (ANOVA) with $P < 0.05$ considered statistically significant.
Data exclusions	No data were excluded.
Replication	All data are repeated for three times to verify the reproducibility of the experimental findings.
Randomization	All samples are randomly grouped.
Blinding	The study was open label.

Reporting for specific materials, systems and methods

We require information from authors about some types of materials, experimental systems and methods used in many studies. Here, indicate whether each material, system or method listed is relevant to your study. If you are not sure if a list item applies to your research, read the appropriate section before selecting a response.

Materials & experimental systems

n/a	Involved in the study	
[ ]	[x]	Antibodies
[ ]	[x]	Eukaryotic cell lines
[x]	[ ]	Palaeontology and archaeology
[ ]	[x]	Animals and other organisms
[x]	[ ]	Clinical data
[x]	[ ]	Dual use research of concern
[x]	[ ]	Plants

Methods

n/a	Involved in the study	
[x]	[ ]	ChIP-seq
[x]	[ ]	Flow cytometry
[x]	[ ]	MRI-based neuroimaging

Antibodies

Antibodies used	Antibodies used in this study were listed as follows:<br>The western blot experiments were performed using primary antibodies against VEGFA (19003-1-AP, Proteintech), HMOX1 (AF5393, Affinity), ITGA2 (DF2540, Affinity), HIF-1A (BF8002, Affinity), TGFBR2 (AF0262, Affinity), Smad3 (9523T, CST), MMP1 (DF6325, Affinity), α-SMA (AF1032, Affinity), elastin (DF7598, Affinity), COL1 (GB11022, Servicebio), COL3 (AF0136, Affinity), fibronectin (ab45688, Abcam).

	The urethral samples were stained for immunofluorescence for epithelial cytokeratin AE1/AE3 (Santa Cruz Biotechnology, Inc.), CD31 (Proteintech Group, Inc.), α-smooth muscle actin (Proteintech Group, Inc.), COL3 (Santa Cruz Biotechnology, Inc.), En1 (Santa Cruz Biotechnology, Inc.), CD206 (Proteintech Group, Inc.), and PCNA (Proteintech Group, Inc.). Nuclei were stained with DAPI (1:500, Life Technologies).
Validation	These antibodies were respectively purchased in Proteintech Group, Inc., Affinity Biotechnology, Inc., Servicebio Biotechnology, Inc., Santa Cruz Biotechnology, Inc. and so on. All these antibodies have been validated for their use in western blot and immunofluorescence staining. All procedures were carefully operated according to the manufacturer's instructions.

Eukaryotic cell lines

Policy information about cell lines and Sex and Gender in Research

Cell line source(s)	Human Umbilical Vein Endothelial Cells (HUVECs) are cells from the vein of umbilical cord. Rabbit Fibroblasts are from the skin of rabbits.
Authentication	HUVECs are tested for specific antibodies against vWF/Factor VIII, CD31 and uptake of Dil-Ac-LDL by immunofluorescent approach. Rabbit Fibroblasts are tested for specific antibodies against α-SMA and vimentin by immunofluorescent approach.
Mycoplasma contamination	Cells are detected negative for the presence of HBV, HCV, mycoplasma, bacteria, yeast and fungi.
Commonly misidentified lines (See ICLAC register)	No commonly misidentified cell lines were used.

Animals and other research organisms

Policy information about studies involving animals; ARRIVE guidelines recommended for reporting animal research, and Sex and Gender in Research

Laboratory animals	Fifteen adult male New Zealand white rabbits with an average body weight of 2.5 kg were used.
Wild animals	All animals were purchased and raised by the animal management of Shanghai Jiao Tong University Affiliated Sixth People's Hospital.
Reporting on sex	Adult male New Zealand white rabbits were used.
Field-collected samples	All animals were carefully raised in the Shanghai Jiao Tong University Affiliated Sixth People's Hospital.
Ethics oversight	All animal experiments were performed in accordance with the guidelines for animal care. The animal protocol (SYXK 2017-0240) was approved by the Institutional Animal Care and Use Committee of the Shanghai Jiao Tong University Affiliated Sixth People's Hospital.

Note that full information on the approval of the study protocol must also be provided in the manuscript.
