# Supplementary material for: Four-dimensional hydrogel dressing adaptable to the urethral microenvironment for scarless urethral reconstruction
Source: Nat Commun. 2023 Nov 22;14:7632. doi: 10.1038/s41467-023-43421-w (PMC10665446; doi:10.1038/s41467-023-43421-w)

TGFBR2

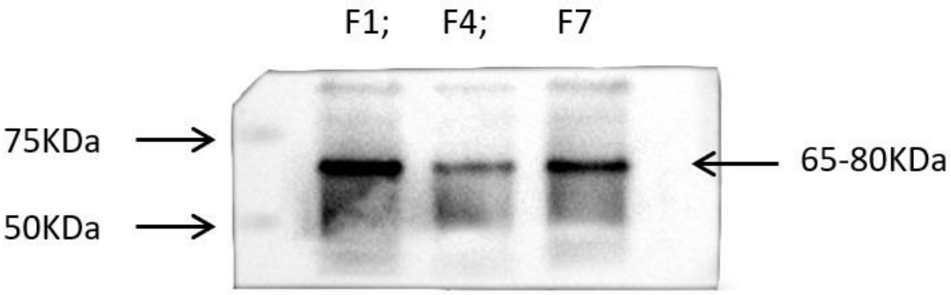

smad3

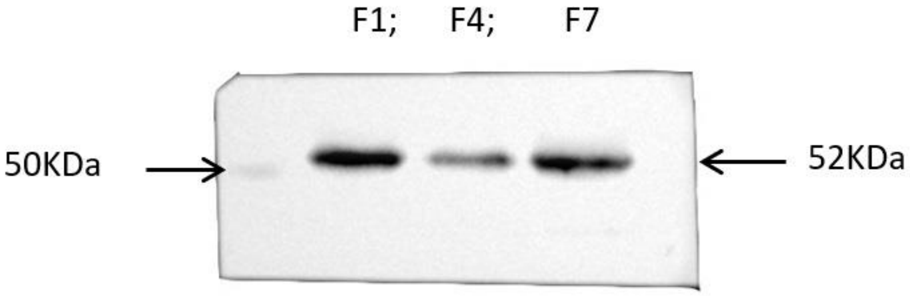

MMP1

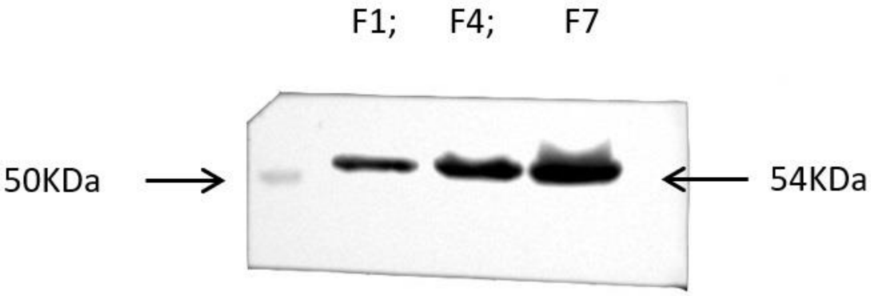

α-SMA

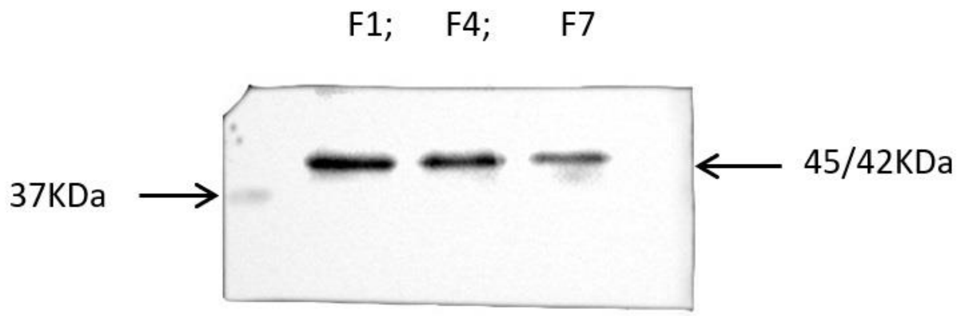

Elastin

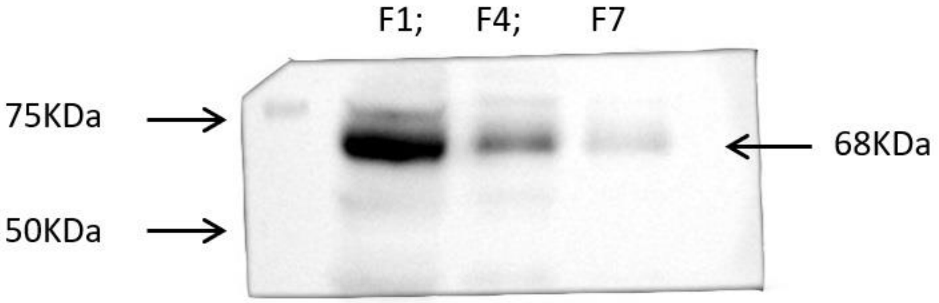

Col I

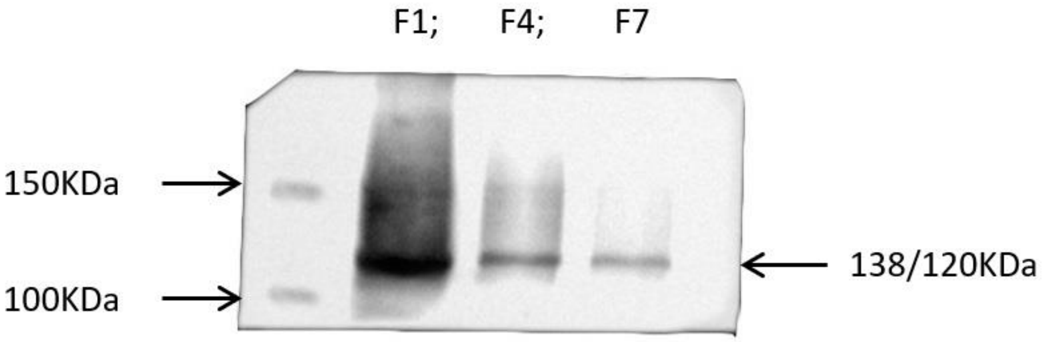

Col 3

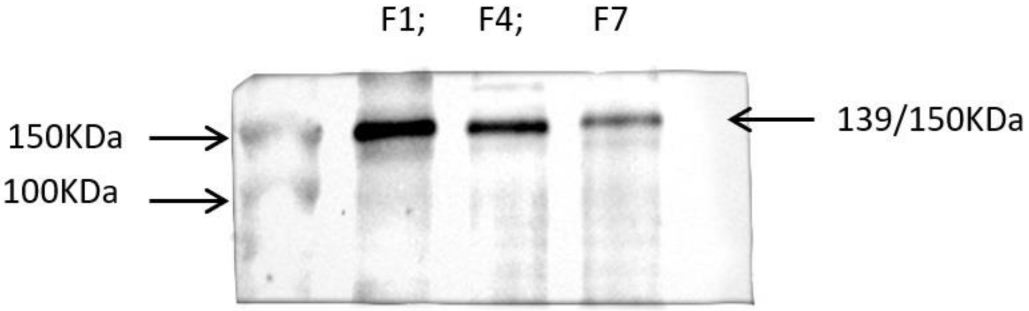

Fibronectin

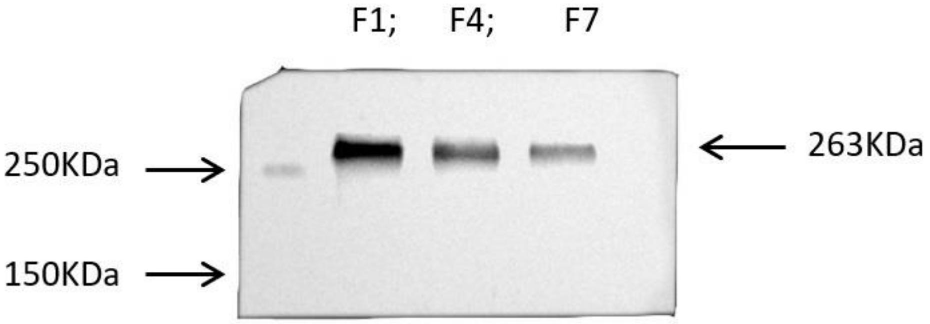

β-actin

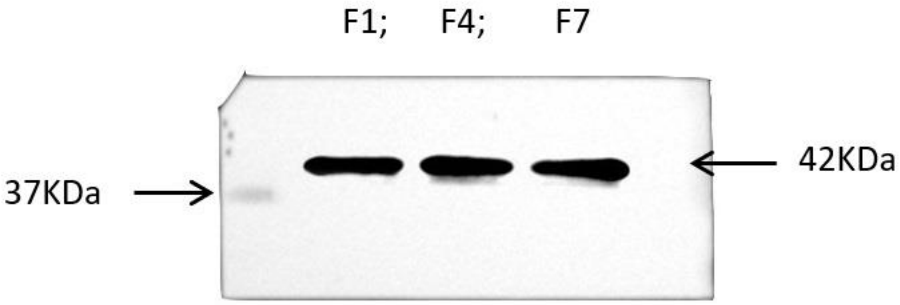

VWF

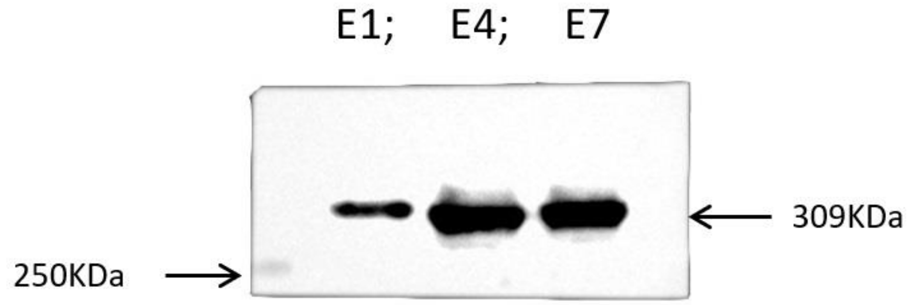

CD31

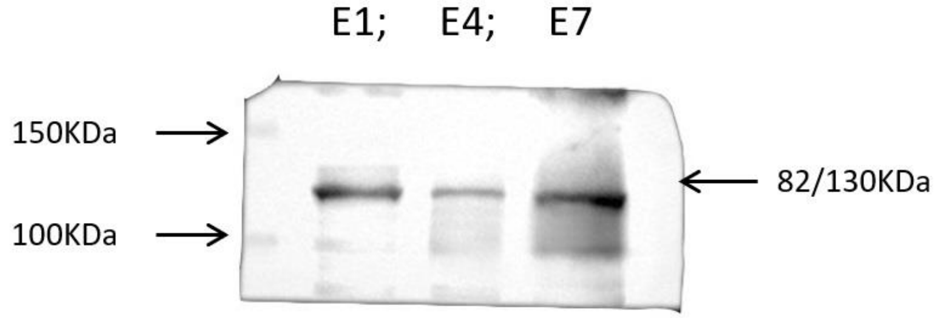

β-actin

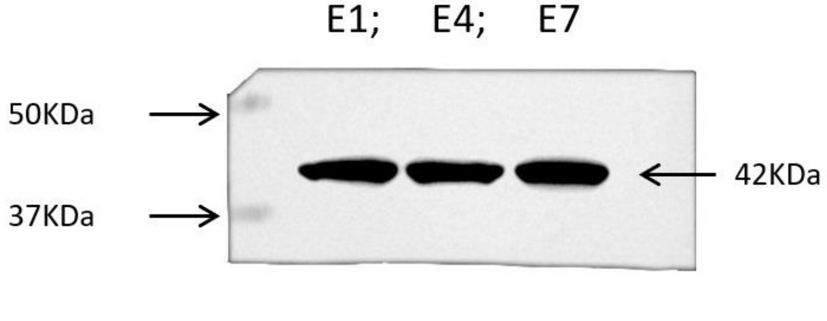

VEGFα

F1; F4; F7

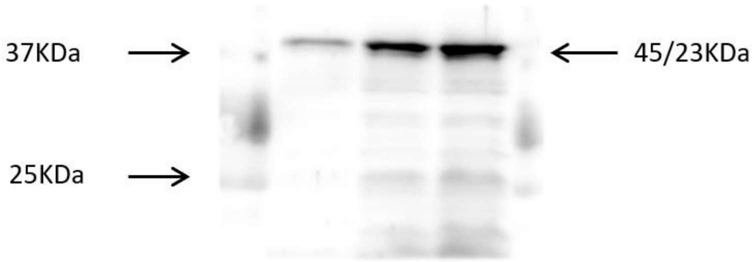

ITGA2

F1; F4; F7

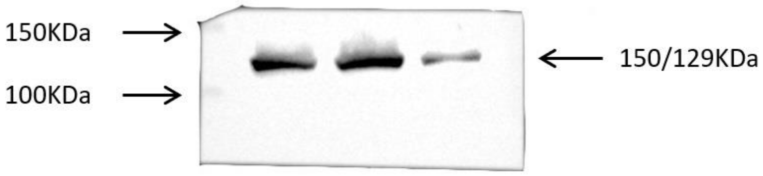

Hif-1α

F1; F4; F7

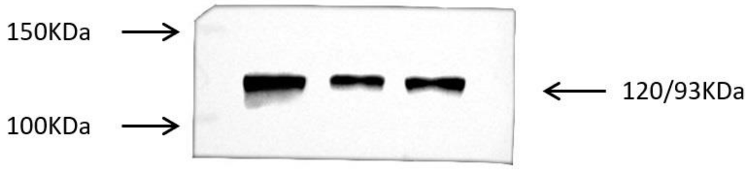

β-actin

F1; F4; F7

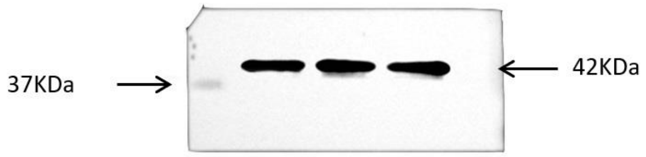

VEGFα

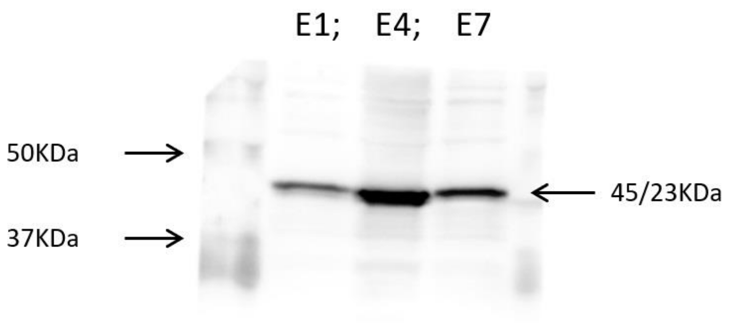

ITGA2

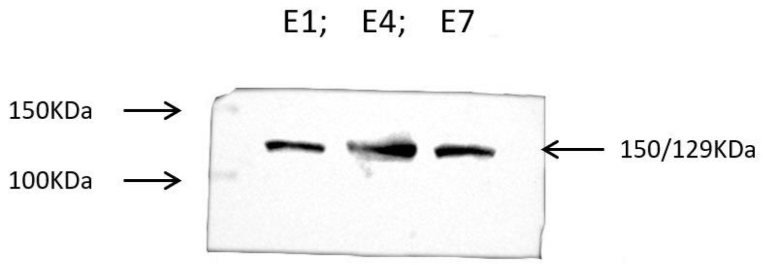

Hif-1α

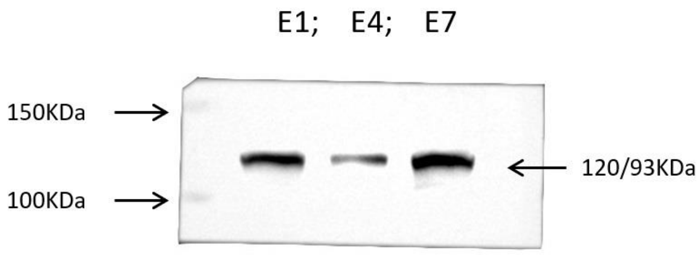

β-actin

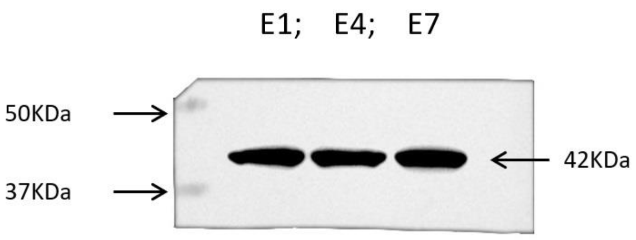

Supplement: Supplementary file 4 — Source Data [file 41467_2023_43421_MOESM4_ESM.zip › Source Data/Supplementary files (WB scans).pdf]
